# Laccase: a multi‐purpose biocatalyst at the forefront of biotechnology

**DOI:** 10.1111/1751-7915.12422

**Published:** 2016-10-03

**Authors:** Diana M. Mate, Miguel Alcalde

**Affiliations:** ^1^ Department of Biocatalysis Institute of Catalysis CSIC Cantoblanco 28049 Madrid Spain

## Abstract

Laccases are multicopper containing enzymes capable of performing one electron oxidation of a broad range of substrates. Using molecular oxygen as the final electron acceptor, they release only water as a by‐product, and as such, laccases are eco‐friendly, versatile biocatalysts that have generated an enormous biotechnological interest. Indeed, this group of enzymes has been used in different industrial fields for very diverse purposes, from food additive and beverage processing to biomedical diagnosis, and as cross‐linking agents for furniture construction or in the production of biofuels. Laccases have also been studied intensely in nanobiotechnology for the development of implantable biosensors and biofuel cells. Moreover, their capacity to transform complex xenobiotics makes them useful biocatalysts in enzymatic bioremediation. This review summarizes the most significant recent advances in the use of laccases and their future perspectives in biotechnology.

## Introduction

Laccases (EC 1.10.3.2, benzenediol:oxygen oxidoreductases) are typically extracellular monomeric glycoproteins that belong to the multicopper oxidase family (Solomon *et al*., 1996). These enzymes catalyse the oxidation of a wide array of compounds coupled to the four‐electron reduction of molecular oxygen to water (Morozova *et al*., 2007a). Laccases are widely distributed in fungi (mostly white‐rot fungi (Brijwani *et al*., 2010), higher plants (Mayer and Staples, 2002) and bacteria (Santhanam *et al*., 2011), having also been reported in lichens (Laufer *et al*., 2009) and sponges (Li *et al*., 2015). Furthermore, polyphenol oxidases with laccase‐like activity have been described in oysters (Luna‐Acosta *et al*., 2010), insect cuticles (Lang *et al*., 2012) and metagenome libraries of bovine rumen (Beloqui *et al*., 2006). The biological function of laccases is determined by their origin and the stage of life of the organism producing them: fungal laccases are involved in stress defence, morphogenesis, fungal plant‐pathogen/host interactions and lignin degradation (Alcalde, 2007); bacterial laccases participate in pigmentation, morphogenesis, toxin oxidation and protection against oxidizing agents and UV light (Singh *et al*., 2011); plant laccases are involved in wound responses and lignin polymerization (Mayer and Staples, 2002); while the role of laccases in lichen physiology remains unknown (Laufer *et al*., 2009).

Laccase substrates include aromatic compounds (such as *ortho*‐ and *para*‐diphenols, methoxysubstituted phenols, diamines and benzenethiols), metal ions (Mn^2+^) and organometallics (e.g. [W(CN)_8_]^4‐^, [Fe(EDTA)]^2‐^) (Alcalde, 2007). Moreover, the scope of laccase substrates can be widened to higher‐redox potential compounds than laccase itself (in some cases bulky and recalcitrant substrates) with the help of diffusible electron carriers (defined as laccase redox mediators) like 2,2′‐azino‐bis(3‐ethylbenzothiazoline‐6‐sulphonic acid) (ABTS) or 1‐hydroxybenzotriazole (HBT) that constitute the laccase‐mediator system (LMS) (Morozova *et al*., 2007b).

The laccase mechanism of action involves two individual sites that bind the reducing substrate and O_2_ with four catalytic copper atoms: the paramagnetic type 1 copper (T1Cu) responsible for the characteristic blue colour of the protein in the reduced resting state, where the substrate oxidation takes place; the T2Cu and the two T3Cu that are clustered 12 Å away from the T1Cu. This trinuclear cluster is where O_2_ is reduced to two molecules of water, receiving four consecutive electrons from four independent mono‐oxidation reactions at the T1Cu site through a strictly conserved His‐Cys‐His electron transfer route (Mot and Silaghi‐Dumitrescu, 2012). To date, about 90 laccase structures have been resolved by X‐ray crystallography, including native and mutant structures, as well as those complexed with substrates, inhibitors and oxidation products (Hakulinen and Rouvinen, 2015).

Laccases are usually classified as low‐, medium‐ or high‐redox potential in function of their redox potential at the T1Cu (E^0′^_T1_) (Mate and Alcalde, 2015). Bacterial and plant laccases form the group of low‐redox potential laccases, with an E^0′^_T1_ <+460 mV versus Normal Hydrogen Electrode (NHE) and with a methionine residue as the T1Cu axial ligand. Fungal laccases include both medium‐ and high‐redox potential enzymes. The medium‐redox potential laccases from ascomycetes and basidiomycetes have an E^0′^_T1_ ranging from +460 to +710 mV versus NHE, and typically with a leucine residue as the non‐coordinating axial ligand. High‐redox potential laccases (HRPLs) are produced by basidiomycete white‐rot fungi, with an E^0′^_T1_ between +730 and +790 mV versus NHE and a phenylalanine as the non‐coordinating axial ligand. From a biotechnological viewpoint, HRPLs generate much interest as their E^0′^_T1_ allows them to oxidize a wider range of substrates than their low‐ and medium‐redox potential counterparts (Rodgers *et al*., 2010).

The potential uses of laccases have been reviewed extensively in recent years, and the reader is referred to other reviews about these enzymes addressing applications in the food industry (Osma *et al*., 2010), in the pulp and paper industry (Virk *et al*., 2012), in the forest product industry (Widsten and Kandelbauer, 2008), in grafting reactions (Kudanga *et al*., 2011), in organic synthesis (Riva, 2006; Kunamneni *et al*., 2008a; Mikolasch and Schauer, 2009; Witayakran and Ragauskas, 2009), in bioremediation (Viswanath *et al*., 2014; Zucca *et al*., 2016) and from the point of view of patents (Kunamneni *et al*., 2008b). In addition, exhaustive reviews have recently focused on the engineering of these enzymes for different biotechnological needs (Mate and Alcalde, 2015; Pardo and Camarero, 2015). The aim of this review is to summarize and update the most significant applications of laccases in biotechnology, centring on the main up‐and‐coming uses of this versatile biocatalyst.

## Laccases in the cutting edge of biotechnology

**Figure 1:**
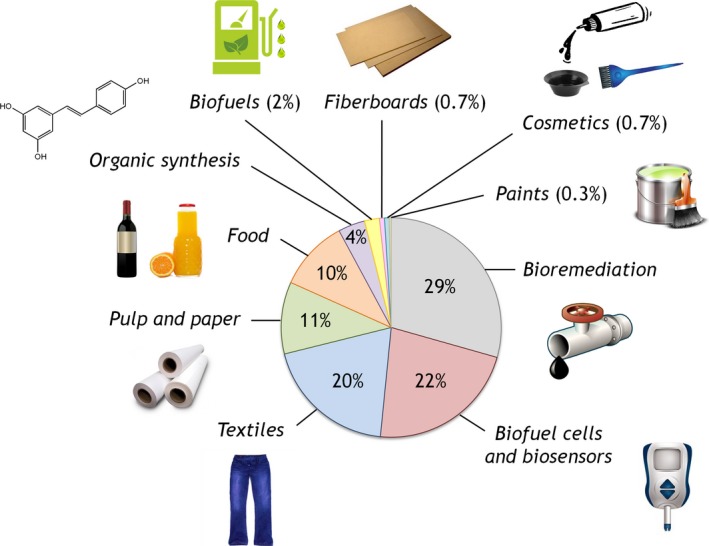
Breakdown of the biotechnological applications of laccases. Data extracted from Scopus database search for articles that included the following keywords: (i) ‘laccase’ and ‘bioremediation’; (ii) ‘laccase’ and ‘biofuel cell’ or ‘biosensor’; (iii) ‘laccase’ and ‘textiles’ or ‘textiles industry’; (iv) ‘laccase’ and ‘pulp and paper’ or ‘pulp and paper industry’; (v) ‘laccase’ and ‘food’ or ‘food industry’; (vi) ‘laccase’ and ‘organic synthesis’; (vii) ‘laccase’ and ‘biofuel production’; (viii) ‘laccase’ and ‘fibreboards’; (ix) ‘laccase’ and ‘cosmetics’; (x) and ‘laccase’ and ‘paints’.

The last decades have witnessed a heightened interest in the use of laccases as biocatalysts to replace conventional chemical processes in the textile, pulp and paper and pharmaceutical industries. These enzymes also have possible applications in other sectors, such as the cosmetic, paint and furniture industries. Additionally, laccases have a place in the production of bioethanol from lignocellulose materials as feedstock. Indeed, the potential use of laccases for industrial and biotechnological purposes is a thriving area of research, as depicted in Fig. 1.

Among the commercially available laccases, we can find bacterial laccases that are heterologously expressed in *Escherichia coli*, as well as laccases from the lacquer tree *Rhus vernicifera*, from filamentous fungi (*Aspergillus* sp.) and from several basidiomycete species including *Agaricus bisporus*,* Cerrena unicolor* and *Trametes versicolor* (Table 1). Moreover, the laccase from the ascomycete *Myceliophthora thermophila* has been adapted for different purposes (see below). In terms of successful commercial products based on laccases, we can find preparations for colour enhancement in tea, cork treatment, pulp bleaching, or denim bleaching and finishing, while many others are in the pipeline (Kunamneni *et al*., 2008b; Piscitelli *et al*., 2013).

**Table 1:** Commercially available laccases

Laccase source	Company	Specific activity (U mg^−1^)	Unit definition
*Agaricus bisporus*	ASA Spezialenzyme GmbH	> 5	Conversion of 1 μmol catechol per minute at pH 6.0 and 25°C
Bacterial origin^a^^,^ b	MetGen	78	Conversion of 1 μmol ABTS per minute at pH 3.0 and 60°C
*Cerrena unicolor*	Jena Bioscience	n.m.	Conversion of 1 μmol ABTS per minute at pH 4.5 and 25°C
*Trametes versicolor*	ASA Spezialenzyme GmbH	> 1	Conversion of 1 μmol syringaldazine per minute at pH 5.0 and 25°C
*Trametes versicolor*	Sigma‐Aldrich	≥ 0.5	Conversion of 1 μmol catechol per minute at pH 5.0 and 25°C
*Trametes versicolor* c	Sigma‐Aldrich	≥ 0.3	Conversion of 1 μmol ABTS per minute at pH 4.5 and 25°C
*Agaricus bisporus*	Sigma‐Aldrich	≥ 4	Conversion of 1 μmol catechol per minute at pH 6.0 and 25°C
*Aspergillus sp*.	Sigma‐Aldrich	> 10^6^	Conversion of 1 mmol of syringaldazine per minute at pH 7.5 and 30°C
*Rhus vernicifera*	Sigma‐Aldrich	≥ 50	ΔA_530_ of 0.001 per minute at pH 6.5 at 30°C in a 3 ml reaction volume using syringaldazine

a The name of the bacterial species is not specified.

b Laccase commercialized as thermoinactivated liquid crude cell lysate.

c Laccase commercialized as cross‐linked enzyme aggregate (CLEA).

n.m.: not mentioned in the specification sheet.

### Food

The food industry makes use of laccases due to their ability to foster homo‐ and heteropolymerization reactions. They can be used in wine and beer stabilization, fruit juice processing, baking, improvement of food sensory parameters and sugar beet pectin gelation. Wines contain a high concentration of phenolic compounds, which affect their taste, colour and gustative sensations. Thus, polyphenols can be selectively removed by laccase to avoid any unwanted modification of the wine's organoleptic properties (Osma *et al*., 2010). Furthermore, laccase from *M. thermophila* is being commercialized for the treatment of cork stoppers for wine bottles (Suberzyme^®^; Novozymes, Bagsværd, Denmark). In this process, the laccase oxidizes phenols and the released phenoxyl radicals undergo non‐enzymatic homopolymerization, avoiding the generation of 2,4,6‐trichloroanisole that is responsible for the cork taste. This oxidative polymerization also modifies the cork's surface, increasing its hydrophobicity and reducing the extraction of substances into the wine (Conrad *et al*., 2000).

Haze formation during long‐term beer storage represents a persistent problem in the brewing industry (Kunamneni *et al*., 2008b). Haze is formed through the interaction between proanthocyanidins (a class of polyphenols present in beer) and specific haze‐active proteins. To resolve this matter, laccases have been used to oxidize polyphenols in beer, the polyphenol complexes formed then being removed by filtration or other separation methods. Laccases have also been used to remove O_2_ at the end of the beer production process in order to enhance beer storage life (Alcalde, 2007). Laccase scavenges O_2_, which would otherwise react with fatty acids, amino acids, proteins and alcohols to form off‐flavour precursors. This application can be found in the commercial preparation Flavourstar^®^ (Novozymes), which is again based on the laccase from *M. thermophila*.

Excessive oxidation of phenolic compounds has been considered detrimental to the organoleptic characteristics of fruit juices (Osma *et al*., 2010). Clear fruit juices are usually stabilized to delay the onset of polyphenol‐protein haze formation, and there are several studies in which fruit juices have been stabilized using laccase. In some cases, laccase treatment of juice resulted in the removal of a high fraction of polyphenols and enhanced stabilization compared with the conventionally treated juice (Cantarelli, 1986). By contrast, some juice treated with laccase showed increased susceptibility to browning during storage, and they were less stable than the physical‐chemically treated juice (Giovanelli and Ravasini, 1993; Gökmen *et al*., 1998; Sammartino *et al*., 1998). It has been reported that the treatment of fruit juices with laccase in conjunction with a filtration process can improve colour and flavour stability (Cantarelli and Giovanelli, 1990; Maier *et al*., 1990; Ritter *et al*., 1992; Stutz, 1993).

Very recently, low‐cost carriers for laccase immobilization have been used in the clarification of fruit juice (Bezerra *et al*., 2015). Specifically, laccase from *T. versicolor* was immobilized in coconut fibres (CF) activated with glutaraldehyde. The laccase‐glutaraldehyde‐CF matrix was used to clarify apple juice, lightening the original juice colour by 61% and removing 29% of its turbidity. A recombinant POXA1b laccase from *Pleurotus ostreatus* was also recently immobilized on epoxy activated poly(methacrylate) beads and tested in the clarification of several fruit juices, producing a reduction in phenol of up to 45% (Lettera *et al*., 2016). Moreover, the laccase‐treated juice had a comparable flavanone content to the non‐treated juice but a dramatic reduction in vinyl guaiacol (an off‐flavour with a pepper‐like aroma).

Laccases have also been investigated in baking to improve dough machinability and the softness of the end‐product (Labat *et al*., 2000), as well as in teas and oil‐containing products to enhance colour and flavour quality, respectively (Bouwens *et al*., 1997; Petersen *et al*., 1999). In addition, the gelling effects of laccases have been studied in blackcurrant juice, luncheon meat and milk with added sugar beet pectin (Norsker *et al*., 2000). In a recent study, the effect of laccase and LMS on stirred milk yoghurt has been assessed in a process that mimics that of industrial production (Mokoonlall *et al*., 2016). The treatment with laccase resulted in protein degradation at the molecular level, while the addition of the natural redox mediator vanillin promoted the formation of higher molecular weight oligomers.

### Textiles

Laccases have attracted increasing interest in the textile industry to be used in processes ranging from the bleaching of denim fabrics (Yavuz *et al*., 2014; Iracheta‐Cárdenas *et al*., 2016) to the enhancement of the whiteness in the conventional peroxide bleaching of cotton (Tzanov *et al*., 2003). In this context, there are more than 19 commercial laccase‐based products for denim bleaching marketed by at least 14 companies around the world (Rodríguez‐Couto, 2012). Laccases can also oxidize various aromatic compounds (including phenols and anilines) to concomitantly promote non‐enzymatic homo‐ and/or hetero‐coupling reactions yielding a colour palette of different valuable dyes for textiles (including phenoxazine and azo dyes) (Polak and Jarosz‐Wilkolazka, 2012; Sousa *et al*., 2013). In particular, laccases have been used to dye cotton and wool fabrics with hetero‐polymeric dyes generated in situ by the oxidative hetero‐coupling of colourless precursors and modifiers initiated by laccase (Hadzhiyska *et al*., 2006; Díaz Blanco *et al*., 2009). Given that the solubility of precursors at acid pH is poor, along with the fact that the non‐enzymatic Michael addition for cross‐coupling requires basic reaction conditions, our laboratory has just developed alkaline laccases from *M. thermophila* to synthesize C‐N heteropolymeric dyes (Torres‐Salas *et al*., 2013 and unpublished material). In another interesting study, the synthesis of substituted phenoxazinones and phenazines dyes from *o*‐phenylenediamines, substituted *p*‐phenylenediamines and *o*‐aminophenols by the CotA laccase from *Bacillus subtilis* and the laccase from *T. versicolor* was described (Sousa *et al*., 2014). The starting aromatic amines were first characterized electrochemically and oxidized by laccase on a preparative scale with good yields (up to 90%) giving rise to novel phenazine and phenoxazinone dyes.

Laccases are also included in cleaning formulations to eliminate the odour on fabrics and the detergents generated during cloth washing (Kunamneni *et al*., 2008b). Indeed, a LMS was applied to reduce the shrinkage of wool (Yoon, 1998). More recently, wool fabrics have been coated with the water insoluble phenolic compound lauryl gallate, using laccase as a grafting biocatalyst (Hossain *et al*., 2009). The functionalization reaction was performed in an 80/20 (v/v, %) aqueous‐ethanol medium, maintaining a compromise between the conditions at which the laccase remains active and those of substrate solubility. This study opens up new possibilities for the development of multifunctional textile materials with antibacterial, antioxidant and water repellent properties.

### Pulp and paper

During industrial paper production, lignin in the wood pulp must be separated and degraded. Traditional delignification procedures involve the use of polluting chlorine‐containing reagents (Virk *et al*., 2012). Thus, for decades, there have been attempts to replace these conventional chlorine‐based delignification processes with cleaner and milder strategies, paying special attention to the pre‐treatment of wood pulp with ligninolytic oxidoreductases. Laccases are preferred to peroxidases (lignin, manganese and versatile peroxidases) for these reactions as the first are fuelled by O_2_ rather than H_2_O_2_ and unlike peroxidases, and their activity is not inhibited by the co‐substrate. Enzymatic bleaching of flax pulp with laccases from different fungi and with redox mediators of natural and synthetic origin has been reported (Camarero *et al*., 2002; Fillat *et al*., 2010). As such, a LMS is currently marketed to increase throughput in mechanical pulping, to enhance paper strength and to reduce pitch problems (MetZyme^®^ LIGNO^™^; MetGen, Kaarina, Finland).

The elimination of the flexographic inks used in printing is a critical aspect of paper recycling. To provide alternative and bio‐based deinking methods, laccases from the ascomycete *M. thermophila* and from the basidiomycetes *Trametes villosa*,* Coriolopsis rigida* and *Pycnoporus coccineus* were tested for decolourization of four flexographic inks in the presence of natural and synthetic mediators (Fillat *et al*., 2012). The three basidiomycete laccases had better decolourization capacities than the *M. thermophila* laccase, accelerating decolourization by using natural and synthetic mediators (especially HBT). Indeed, only the lignin‐derived mediators acetosyringone and methyl syringate were able to decolourize all the inks assayed with *M. thermophila* laccase, although all activity was lost after 4 h.

Laccase‐induced grafting of phenols to flax fibres for paper production has also been described recently (Aracri *et al*., 2010; Fillat *et al*., 2012). However, the treatment of flax and sisal pulps with laccases from *P. cinnabarinus* and *T. villosa* was evaluated in the presence of different phenolic compounds (Aracri *et al*., 2010). In most cases, laccase treatment led to the covalent incorporation of the phenols into the fibres, with the highest extent of phenol grafting observed when *p*‐hydroxycinnamic acids, *p*‐coumaric and ferulic acids were used. Conversely, the treatment of unbleached flax fibres with laccase from *P. cinnabarinus* and low‐molecular‐weight phenols was assessed (Fillat *et al*., 2012). Paper handsheets from pulps treated with laccase and phenol were evaluated for their antimicrobial and optical properties, showing antimicrobial activity against the three bacterial species tested (*Staphylococcus aureus*,* Pseudomonas aeruginosa* and *Klebsiella pneumoniae*), as well as a decrease in brightness and an increase in colouration.

### Biofuels

Lignocellulosic materials are among the most promising feedstocks for bioethanol production. However, their utilization depends on the efficient hydrolysis of polysaccharides, which needs a cost‐effective pre‐treatment of biomass to remove lignin and expose sugars to hydrolytic enzymes. Laccases play a key role in lignin biodegradation, and therefore, their potential use as agents in biofuel production is being studied, and as biocatalysts to remove yeast growth inhibitors (mainly phenolics) for the subsequent enzymatic processes (Kudanga and Le Roes‐Hill, 2014). A laccase from *T. versicolor* heterologously expressed in *Saccharomyces cerevisiae* improved the production of bioethanol by eliminating phenolic compounds (Larsson *et al*., 2001). More recently, a newly identified laccase from the white‐rot fungus *Ganoderma lucidum* was assessed to detoxify lignocellulosic hydrolysates and in bioethanol production. This laccase removed 84% of the phenolic content in corn stover hydrolysate, and when added prior to cellulase hydrolysis, it improved ethanol yield by 10% (Fang *et al*., 2015).

An interesting new trend in this developing field is the engineering of a full consolidated bioprocessing microbe (by engineering an artificial secretome in yeast) that contains the key enzymes of the ligninolytic consortium (Gonzalez‐Perez and Alcalde, 2014; Alcalde, 2015).

### Organic synthesis

Laccases are useful biocatalysts for the pharma sector since they can catalyse a wide range of synthetic reactions, ranging from the transformation of antibiotics to the derivatization of amino acids for the synthesis of metabolically stable amino acid analogues (Piscitelli *et al*., 2012). As such, laccases have been used to synthesize complex medical products, such as anti‐cancer drugs (e.g. vinblastine, mitomycin and actinomycin), immunosuppressors (e.g. cyclosporin A) and antibiotics (e.g. penicillin X dimer and cephalosporins) (Kunamneni *et al*., 2008a and references therein). In addition, laccases have been used to oxidize the steroid hormone 17β‐estradiol and stilbenic phytoalexin *trans*‐resveratrol, generating dimers or oligomers after coupling of the radical intermediates (Nicotra *et al*., 2004a,b). Moreover, they have also been employed in the enzymatic derivatization of amino acids, such as L‐tryptophane, L‐phenylalanine or L‐lysine (Mogharabi and Faramarzi, 2014).

Catechin polymers can be used to attenuate postprandial hypercholesterolaemia and hyperlipidaemia, and their synthesis can be catalysed by *M. thermophila* laccase (Denilite^®^ IIS; Novozymes), influencing lipid and cholesterol absorption (Jeon and Imm, 2014). Laccase‐catalysed catechin polymers have stronger inhibitory activity against pancreatic lipase and cholesterol esterase than the catechin monomer. Another potential application of laccases is in the oxidation of iodide to generate iodine (I_2_), an inexpensive and efficient antimicrobial compound (Xu, 1996). Iodide oxidation by laccase was proposed to inactivate *Bacillus anthracis* spores (Niederwöhrmeier *et al*., 2008), and in the past year, the laccase‐mediated synthesis of I_2_ based on an artificial neural network was studied with a genetic algorithm (Schubert *et al*., 2015).

The tandem use of laccases and lipases has been described in the synthesis of enantiomerically enriched dimeric phenols with structures similar to the β‐5 dimers found in lignin (Gavezzotti *et al*., 2011). Laccase from *T. versicolor* was used to oxidize the commercially available isoeugenol, and the two resulting enantiomers were separated by alcoholysis cleavage using a lipase. This process led to the isolation of the target compounds with an *ee* of up to 90%. Interestingly, laccases have also been shown to be able to oxidize alcohols in combination with palladium catalysts (Mekmouche *et al*., 2015). Specifically, the LAC3 laccase from *Trametes* sp. C30 was combined with four different water‐soluble palladium complexes known to oxidize primary and secondary alcohols under harsh conditions (high temperature and pressure). The laccase‐palladium complexes were then evaluated for the aerobic oxidation of veratryl alcohol into veratryl aldehyde at room temperature and atmospheric pressure. As a result, the association of the laccase and the palladium (II) complexes tested improved the catalytic efficiency of the complex up to seven fold.

### Cosmetics

The oxidative potential of laccases has also been harnessed in the cosmetic sector for the manufacturing of personal‐care products. While cosmetic and dermatological preparations containing laccases were patented for skin lightening (Golz‐Berner *et al*., 2004), it is in the field of hair bleaching/dying where laccases have broader applications. The bleaching and/or dying of hair usually involve the use of harsh chemicals like H_2_O_2_ that can damage hair and irritate the scalp (Morel and Christie, 2011). Laccases can be used to replace H_2_O_2_ as oxidizing agent in the formulation of hair dyes. As such, novel laccases from the actinomycete *Thermobifida fusca* and from the basidiomycete *Flammulina velutipes* have recently been tested in the oxidation of dye intermediates widely used in hair colouring (Saito *et al*., 2012; Chen *et al*., 2013). Moreover, a hair colour was recently developed comprising butein and: (i) either a combination of a peroxidase with either H_2_O_2_ or a H_2_O_2_ generator; or (ii) a laccase (Bhogal *et al*., 2013).

### Paints

Alkyd resins are polyesters synthesized by the polymerization of polyalcohols, dicarboxylic acids or anhydrides and unsaturated fatty acids (Gooch, 2002). These resins are mainly used as binding agents in coatings, although they also find applications as road markings, house and decorative paints. Chemical drying of these resins is based on heavy‐metal catalysed cross‐linking of the unsaturated fatty acids. Currently, procedures to replace heavy‐metal‐based catalysts with less toxic and environmentally friendlier alternatives are under development. It was recently shown that LMS can effectively replace heavy‐metal catalysts and cross‐link the alkyd resins (Greimel *et al*., 2013). Interestingly, the biocatalytic reaction worked both in aqueous media and in a solid film.

### Furniture

Medium‐density fibreboards (MDF) are dry formed panel products manufactured by cross‐linking of lignocellulosic materials with a synthetic resin under heat pressure in the presence of moisture (Maloney, 1993). They are used to construct a wide variety of furniture such as wardrobes, cupboards, tables, desk tops, TV tables, beds and sofas. Traditional MDF manufacturing was based on non‐enzymatic cross‐linking using polluting compounds like formaldehyde. Currently, concerns about formaldehyde emissions and the increasing prices of petrochemical resins have led to growing interest in enzymatic binder systems as eco‐friendly alternatives to glue lignin‐based materials. LMS has been used to activate lignin on wood fibre surfaces in the pilot‐scale production of MDF (Euring *et al*., 2011). Additionally, a hot‐air/hot‐steam process for the production of wood fibre insulation boards was described that uses a LMS as a naturally based bonding system (Euring *et al*., 2015). Wood fibre insulation boards were glued with the LMS and compared to reference boards prepared with an inactivated LMS, a laccase alone or containing a polymeric glue commonly used during the dry‐process. The boards were then hardened with a steam‐air mixture, with hot‐air, or with hot‐air/hot‐steam, the latter displaying better physical and technical properties than those hardened with steam‐air mixture or hot‐air alone.

### Nanobiotechnology and biomedicine

Due to their ability to catalyse direct electron transfer, laccases have been studied for years in relation to the development of biofuel cells and biosensors (Rodríguez‐Delgado *et al*., 2015). In terms of biosensors, laccases reduce O_2_ to H_2_O and the biosensor then records the oxygen consumption during analyte oxidation. Laccase‐based sensors have been used widely in the food industry to detect polyphenols in fruit juices, wine and teas and to quantify fungal contamination in grape musts (Zouari *et al*., 1988; Ghindilis *et al*., 1992; Cliffe *et al*., 1994; Di Fusco *et al*., 2010). Laccase‐based biosensors have also been developed in biomedicine to detect insulin, morphine and codeine (Bauer *et al*., 1999; Milligan and Ghindilis, 2002).

**Figure 2:**
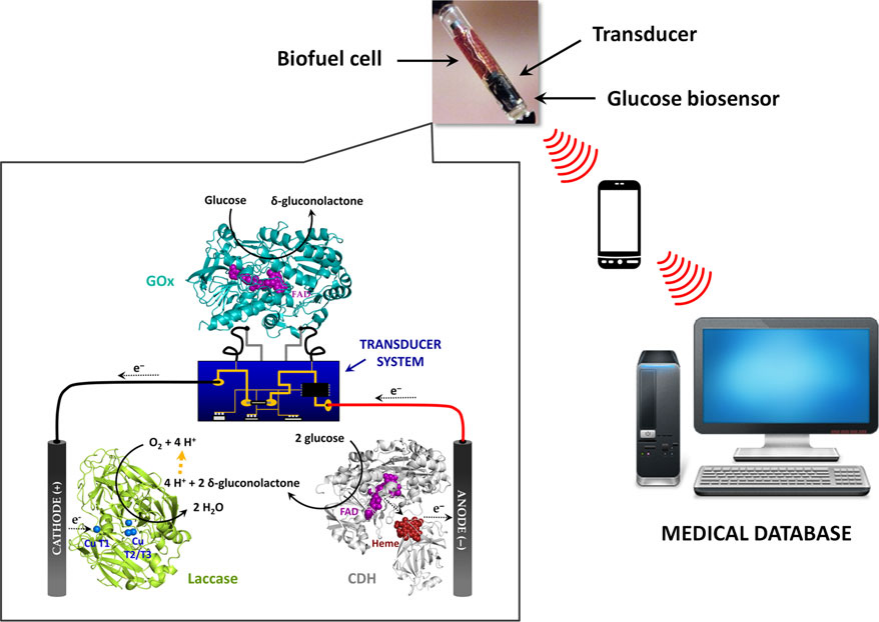
Schematic representation of a self‐contained biodevice to monitor glucose. Glucose oxidase (GOx) is immobilized on the biosensor surface while fungal laccase on the cathode and cellobiose dehydrogenase (CDH) on the anode. CDH oxidizes glucose to δ‐gluconolactone and the electrons are directly transferred via the haem group to the anode. Incoming electrons are then transferred from the cathode via the copper centres to molecular oxygen which is reduced to water.

One of the most exciting applications of HRPLs is their utilization as cathode enzymes in biofuel cells for biomedical purposes (Falk *et al*., 2013) (Fig. 2). Our group was recently successful in evolving the laccase from the basidiomycete PM1 (E^0′^_T1_ = +759 mV versus NHE) to yield a laccase active in human blood (Mate *et al*., 2013). This blood tolerant laccase was then incorporated into a self‐powered and wireless device, opening promising perspectives for applications not only in medical devices but also in environmental monitoring, high‐tech industry and biocomputing (Falk *et al*., 2014). Besides, this laccase mutant was also attached to a low‐density graphite electrode for the oxidation of water at pH > 7, reverting the natural activity of the laccase and opening a thrilling area of research into water splitting (Pita *et al*., 2014).

The development of implantable biofuel cells harvesting power from natural sources is of great interest in nanobiotechnology today. Very recently, preliminary results were published of an enzyme biofuel cell operating in an orange in vivo (MacVittie *et al*., 2015). Specifically, the biofuel cell was composed of catalytic electrodes with glucose dehydrogenase and fructose dehydrogenase immobilized on the anode and with laccase from *T. versicolor* on the cathode. The cathode/anode pair was implanted in orange pulp, extracting power from its content (the glucose and fructose in the juice). In turn, the power harvested from the orange was utilized to supply a wireless electronic system.

A recent trend in biomedical and biomaterial research is the development of polymers with bioresponsive properties to detect potentially pathogenic microorganisms. Bioresponsive hydrogels based on carboxymethylcellulose and peptidoglycan were designed to detect lysozyme in infected wound fluids and cellulases secreted by potentially pathogenic microorganisms, respectively (Schneider *et al*., 2012). A laccase from *Trametes hirsuta* was chemically modified with polyethyleneglycol or methacrylic groups, and it was incorporated into the hydrogels to enhance the signal and the stability relative to simple dye release‐based systems.

### Enzymatic bioremediation

Environmentally hazardous xenobiotic compounds like polycyclic aromatic hydrocarbons (PAHs), phenols and organophosphorus insecticides are known to have teratogenic and carcinogenic effects. These persistent chemicals represent major contaminants of soils and waters, and accordingly, their removal is a priority for most environmental agencies (Alcalde *et al*., 2006; Viswanath *et al*., 2014). Leaving aside microbial bioremediation, the use of laccases in enzyme bioremediation has generated much interest, both in the presence and in the absence of redox mediators. As a rule of thumb, laccase or LMS can oxidize the xenobiotic to release a less toxic product with greater bioavailability, which can be more readily removed by physical and/or mechanical procedures. Examples of this include the removal of PAHs like anthracene or benzopyrene (Majcherczyk *et al*., 1998; Cañas *et al*., 2007; Zumárraga *et al*., 2007; Zeng *et al*., 2016), recalcitrant dyes like Reactive Black 5 or crystal violet (Camarero *et al*., 2005; Wang *et al*., 2016) and organophosphorous compounds, such as the nerve agents VX or Russian VX (Amitai *et al*., 1998). Moreover, oestrogenic hormones found in effluents from sewage treatment can be oxidized by laccases. Indeed, the oxidation of the oestrogens estrone, 17β‐estradiol and 17α‐ethynylestradiol by a fungal laccase from *Trametes* sp. Ha1 has been described (Tanaka *et al*., 2009). Besides, a treatment system was developed that comprised the laccase and a β‐D‐glucuronidase to degrade the 17β‐estradiol 3‐(β‐d‐glucuronide), efficiently eliminating this compound and its intermediate 17β‐estradiol.

## Concluding remarks

From a historic perspective, the study of laccases dates back to the late 19th century, and accordingly, one might reasonably think it as an ‘old–fashioned’ enzyme. Paradoxically, well into the 21st century, the laccase is currently considered a ‘trendy’ enzyme and by many, the ideal green catalyst. However, although several companies offer laccases in their catalogues to the food, textile, pulp and paper, pharma, cosmetic, paint or furniture industries, to fully realize the potential of laccases to compete in the biotechnology race, some hurdles must still be overcome. Thereby, we have to produce laccases in industrially relevant hosts (typically filamentous fungi like *Aspergillus sp*.), as well as at competitive prices and high titres (several g l^−1^). Although some progress has been achieved in this respect through protein engineering (Mate and Alcalde, 2015), the employment of laccases (especially HRPLs) on a large industrial scale is still not the norm. Another important issue is the high cost of redox mediators and their inhibitory potential on laccase activity. In this respect, implementing LMS based on natural mediators derived from lignin combustion is an area worthy of being studied in depth. Last but not least, the design of laccases with customized features through protein engineering will expand the portfolio of highly efficient enzyme variants and their versatility in a range of biotechnology applications, from organic synthesis to that of biofuels and beyond.

## Conflict of interest

The authors have no conflict of interests to declare.
